# Giant conductivity of mobile non-oxide domain walls

**DOI:** 10.1038/s41467-021-24160-2

**Published:** 2021-06-25

**Authors:** S. Ghara, K. Geirhos, L. Kuerten, P. Lunkenheimer, V. Tsurkan, M. Fiebig, I. Kézsmárki

**Affiliations:** 1 Experimental Physics V, Center for Electronic Correlations and Magnetism, University of Augsburg, Augsburg, Germany; 2 Department of Materials, ETH Zurich, Zurich, Switzerland; 3 Institute of Applied Physics, Chisinau, Republic of Moldova

**Keywords:** Ferroelectrics and multiferroics, Ferroelectrics and multiferroics

## Abstract

Atomically sharp domain walls in ferroelectrics are considered as an ideal platform to realize easy-to-reconfigure nanoelectronic building blocks, created, manipulated and erased by external fields. However, conductive domain walls have been exclusively observed in oxides, where domain wall mobility and conductivity is largely influenced by stoichiometry and defects. Here, we report on giant conductivity of domain walls in the non-oxide ferroelectric GaV_4_S_8_. We observe conductive domain walls forming in zig-zagging structures, that are composed of head-to-head and tail-to-tail domain wall segments alternating on the nanoscale. Remarkably, both types of segments possess high conductivity, unimaginable in oxide ferroelectrics. These effectively 2D domain walls, dominating the 3D conductance, can be mobilized by magnetic fields, triggering abrupt conductance changes as large as eight orders of magnitude. These unique properties demonstrate that non-oxide ferroelectrics can be the source of novel phenomena beyond the realm of oxide electronics.

## Abstract (web summary)

Conductive domain walls have been usually found in ferroelectric oxides. Here, the authors report on giant conductivity of domain walls and their magnetically avalanche-like expulsion event in non-oxide ferroelectric GaV_4_S_8_, extending the source of phenomena beyond the realm of oxide electronics.

## Introduction

In order to minimize the energy of a ferroic material, energetically equivalent domain states can form with different orientations of the ferroic order parameter. The number of domain states is dictated by the symmetry reduction upon the para-to-ferroic transition and the population of the domains can be controlled by the conjugate field^1,2^. Domain walls (DWs) are interfaces between adjacent domains, which can host spatially confined states with novel functionalities forbidden in bulk^3,4^. Notable examples of such properties emerging at DWs include spatially confined magnetic states in TbMnO_3_^5^ and GaV_4_Se_8_^6^ as well as local electronic properties in nonpolar ferroelastics, such as ferroelectricity in *A*TiO_3_ (*A* = Sr and Ca)^7,8^ and flexopiezoelectricity in WO_3_^9^.

An intriguing aspect of DWs in ferroelectric insulators is their electrical conductivity, often originating from the discontinuity of the normal component of the polarization across DWs ^10–14^. Such conducting DWs have been observed in several materials, like BaTiO_3_^15^, LiNbO_3_^16,17^, *h*-*R*MnO_3_ (*R* = Er and Ho)^18,19^, and BiFeO_3_^20–22^.

All ferroelectrics in which conductive DWs have so far been reported are oxides, where DW conductivity usually requires a specific strain configuration of the crystal, an improper character of the ferroelectricity or other unusual properties^1,15,18,23–25^ which render the walls immobile and thus curtail their usefulness and flexibility. Additionally, oxide materials are prone to defects, often hampering the utility of their conducting DWs.

In this study, we report on highly conductive DWs in a non-oxide ferroelectric, GaV_4_S_8_, investigated by macroscopic transport studies as well as by piezoresponse force microscopy (PFM) and conductive atomic force microscopy (c-AFM). This compound belongs to the lacunar spinel family, where the V_4_ molecular clusters have been reported to accommodate different number of 3*d* electrons^26–32^. The heterovalent substitution of Ga^3+^ by Ge^4+^ or by Zn^2+^ has been demonstrated to respectively increase or decrease the number of 3*d* electrons occupying the V_4_ molecular units^31^. The stability of the two line compounds, GaV_4_S_8_ and GeV_4_S_8_, both categorized as narrow-gap molecular Mott insulators^32–34^, also points to the high flexibility in the valency of the V_4_ clusters. This multi-valency may provide an intrinsic mechanism to screen charged DWs via mobile carriers, either conduction electrons or holes, without requiring defects and off-stoichiometry. This intrinsic screening process would naturally make charged DWs also conductive. Hence, this material possesses several ingredients that are crucial to realize ideal DW functionalities and are not found to coexist in any oxide material. For example, we found that such charged DWs govern the overall conductivity of this material even though they constitute only a minor fraction of the 3D bulk volume. Furthermore, a “digital” alternation of conducting head-to-head (HH) and tail-to-tail (TT) segments occurs on the nanoscale within the conductive DWs. This may imply the coexistence of both electron- and hole-conduction channels in close proximity, unprecedented in oxide materials. Finally, this compound is a proper ferroelectric in which conductive DWs emerge in the unstrained crystal spontaneously, such that they retain their flexibility, facilitating their electric and magnetic control. We demonstrate that the in situ magnetic erase of DWs, which takes place through an avalanche-like DW expulsion process, changes the conductance of the material by up to eight orders of magnitude.

**Fig. 1:**
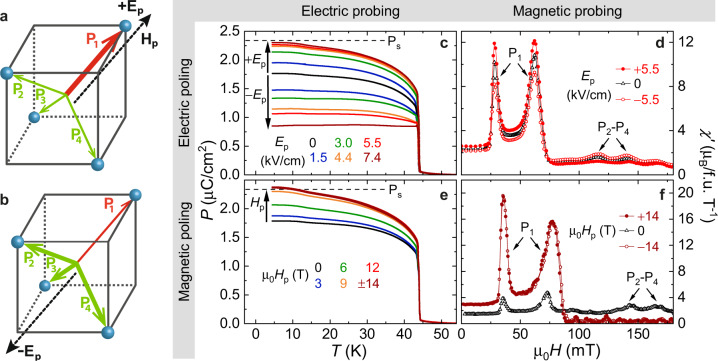
Electric and magnetic control/readout of multiferroic domain population in GaV_4_S_8_. **a**, **b** Schematics about the domain selection process by electric (*E*_*p*_) and magnetic (*H*_*p*_) poling fields applied along the [111] axis. Polarizations of the four domain states, P_1_–P_4_, are indicated with respect to the undistorted V_4_ tetrahedron of the cubic phase. Thick/thin arrows correspond to domains favored/unfavoured by the poling. **c**, **d** Temperature-dependent polarization/field-dependent susceptibility at 11.5 K and their variation with *E*_*p*_. **e**, **f** Temperature-dependent polarization/field-dependent susceptibility at 11.5 K and their variation with *H*_*p*_. In all cases polarization along the [111] axis and susceptibility for *H*∥[111] were recorded.

GaV_4_S_8_ undergoes a ferroelectric transition from a cubic ($F\bar{4}3m$) to a polar rhombohedral (*R*3*m*) phase at *T*_*JT*_ ≃ 45 K, due to a cooperative Jahn–Teller effect lifting the degeneracy of the V_4_ cluster orbitals^27,28^. Since the high-temperature crystal structure already lacks the inversion symmetry, the cubic-to-rhombohedral deformation necessarily leads to the onset of electric polarization. The ferroelectric nature of the rhombohedral phase has been demonstrated by pyroelectric current measurements^35,36^, PFM^37,38^, lattice dynamics studies^32,39^ and by dielectric spectroscopy^40^, as well as supported theoretically^41,42^. The breaking of the $\bar{4}$ symmetry upon the para- to ferroelectric transition results in four polar domain states (P_1_–P_4_) with the polarization vectors along the four 〈111〉-type cubic body diagonals, as shown in Fig. 1a, b. The polarizations of the four domains span 109^∘^ with each other, thus, no ±*P* domains can form in a single crystal^2,27,37,43^. Lamellar domain patterns, formed of two alternating ferroelectric domains and separated by {100}-type mechanically and electrically compatible 109^∘^ DWs, were observed by PFM^37,38^.

Within the polar phase, GaV_4_S_8_ undergoes a magnetic ordering transition at *T*_*C*_ = 13 K to a cycloidal (Cyc) state and subsequently to a ferromagnetic (FM) state below 6 K^43–45^. By magnetic fields in the sub-Tesla range, the Cyc state is turned to a Néel-type skyrmion lattice (SkL) and then to the FM state. The critical fields strongly depend on the orientation of the field with respect to the polar axis, being also the magnetic easy axis^43^. In addition to the polarization induced by the rhombohedral distortion, all the three magnetic phases exhibit sizable magnetoelectric polarizations^35,36^.

## Results and discussion

### Domain wall control and imaging

Figure 1 shows how the multiferroic nature of GaV_4_S_8_ is exploited to control the population of domains by either electric (*E*_*p*_) or magnetic (*H*_*p*_) poling fields and to probe their volume fractions via either electric or magnetic properties. As illustrated in Fig. 1a, b, +*E*_*p*_ applied along the [111] axis favors the P_1_ domain with polarization along the field direction, resulting in the P_1_ mono-domain state. However, when applying −*E*_*p*_, the material acts as a “poling-diode” due to the lack of inversion domains: Upon the suppression of the P_1_ domain state, a P_2_–P_3_–P_4_ multi-domain state is created. Figure 1c, d, respectively, display the temperature-dependent electric polarization along the [111] axis and the field-dependent magnetic susceptibility at 11.5 K for magnetic fields parallel to the [111] axis, both measured after different poling runs with *E*_*p*_∥[111] (for details of the poling protocol see the Methods part). The unpoled state is dominated by the P_1_ domain, as reflected by the large positive value of the polarization (~1.75 *μ*C/cm^2^). This domain imbalance, likely arising from internal strains within the crystal, is further supported by the larger magnitudes of two low-field peaks at ~30 and ~70 mT, as compared to the two high-field anomalies at ~120 and ~140 mT. The former two are associated with the subsequent Cyc → SkL → FM transitions within the P_1_ domain with magnetic easy axis parallel to the magnetic field, while the latter two indicate the same transitions taking place simultaneously in the other three domains with easy axes oblique to the field^43^. Poling with +*E*_*p*_ enhances the polarization, which saturates at *P*_*s*_ ≈ 2.3 μC/cm^2^ for *E*_*p*_ > +4.4 kV/cm, resulting in the P_1_ mono-domain state. In contrast, −*E*_*p*_ decreases the measured polarization, indicating an enhanced volume fraction of the P_2_, P_3_, and P_4_ domains. When the P_1_ domain is fully suppressed, the polarization should reach the value of −*P*_*s*_/3 ≈ −0.8 μC/cm^2^. Thus, the smallest experimentally obtained value of +0.7 μC/cm^2^, recorded after poling with −*E*_*p*_ = 7.4 kV/cm, still points to a multi-domain state formed of all the four domain states. In parallel to the electric-field-driven promotion (+*E*_*p*_) or suppression (−*E*_*p*_) of the P_1_ domain, the two low-field peaks in the susceptibility are enhanced or suppressed, respectively, contrary to the two high-field anomalies.

Due to the coincidence of the magnetic easy axis with the polar axis^43,46^, the domain population can be efficiently controlled also by magnetic field. Poling with *H*_*p*_ along the [111] axis is expected to promote the P_1_ mono-domain state, irrespective of the sign of *H*_*p*_, since the anisotropy energy is an even function of *H*_*p*_. Indeed, Fig. 1e shows a continuous increase of polarization with increasing *H*_*p*_ and its saturation for $|{\mu }_{0}{H}_{p}|\,\ge$ 9 T, when the P_1_ mono-domain state is achieved. The domain population, as probed via the magnetic susceptibility, shows the same trend in Fig. 1f.

**Fig. 2:**
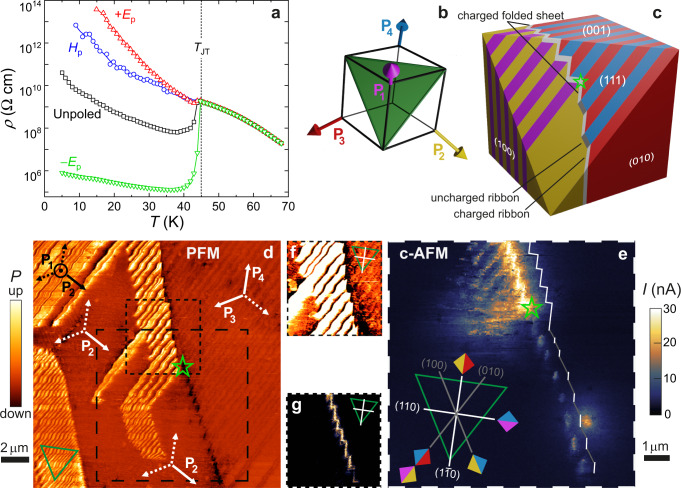
Macro- and microscopic signatures of conductive DWs in GaV_4_S_8_. **a** Dependence of the resistivity on electric (*E*_*p*_ = ±5.5 kV/cm) and magnetic (*μ*_0_*H*_*p*_ = 14 T) poling below *T*_*JT*_, implying the presence of conducting DWs. **b** Orientation of the polarization in the four polar domains, P_1_–P_4_, with respect to the (111) plane (green triangle), which was imaged by PFM and c-AFM. **c** Schematic representation of the domain pattern observed in (**d**–**g**). The crystal orientation and the coloring of the domains are according to (**b**). White and gray DW segments indicate charged (TT or HH) and uncharged HT junctions, respectively. **d** PFM image recorded at 15 K on the (111) surface of an unpoled sample. The polarization directions for domains present/absent in the local domain patterns are indicated by solid/dashed arrows with labeling and orientation according to (**b**). **e** The c-AFM image, recorded over the region with the thick dashed frame in (**d**), confirms the conducting nature of the ribbon- and folded sheet-like DWs, where the folded sheet is a hybrid of alternating HH and TT nanoscale conducting segments, located below and above the green star, respectively. The schematic inset indicates the orientations and assignments of the DWs observed in (**d**, **e**) as well as in the zoomed-in color-thresholded PFM and c-AFM images of (**f**, **g**), both corresponding to the region with the thin dashed frame in (**d**). Orientation and color coding follows those in (**b**, **c**).

Besides controlling and probing the volume fraction of the multiferroic domains, we investigated the influence of the domain population and DW density on the charge transport. The temperature-dependent resistivity in the mono-domain state, as obtained by poling with either +*E*_*p*_ or *H*_*p*_, follows a typical semiconducting behavior both above and below the structural transition with only a tiny anomaly at *T*_*JT*_ (see Fig. 2a). In contrast, the resistivity drops about four orders of magnitude at *T*_*JT*_ in the multi-domain state, realized by poling with −*E*_*p*_, and it is nearly independent of temperature below *T*_*JT*_, implying the presence of delocalized charge carriers. Consequently, the low-temperature resistivity shows a huge difference between the mono- and multi-domain states, reaching eight orders of magnitude at *T* = 15 K. We attribute the enhanced conductivity of the multi-domain state to the presence of conductive DWs. With a smaller drop of the resistivity at *T*_*JT*_, the unpoled crystal represents an intermediate case with a lower density of conductive DWs. This is in agreement with results in Fig. 1, which show the dominance of the P_1_ domain over coexisting P_2_, P_3_, and P_4_ domains in the unpoled crystal.

To identify the type of conductive DWs and to reveal the microscopic mechanism of DW conductivity, we carried out simultaneous PFM and c-AFM studies. Figure 2d, e, respectively, shows a PFM and a c-AFM image, recorded at 15 K on an as-grown (111) surface of an unpoled GaV_4_S_8_ crystal. Since the out-of-plane component of the piezoelectric effect is imaged, only the P_1_ domain with polarization normal to this plane is distinguishable in Fig. 2d^37^. The other three domains, with polarizations all spanning 19^∘^ with the (111) plane, have the same out-of-plane piezoelectric signal. In the rhombohedral phase of GaV_4_S_8_, nine types of mechanically compatible DW can emerge: Three {100}-type uncharged and six {110}-type charged DWs (see Supplementary Fig. 1). On the (111) surface, we observed both {100}-type uncharged and {110}-type charged DWs, as shown in Fig. 2 and described in the following. The most obvious DW structure, seen on the left side of the PFM image, is a lamellar pattern of alternating brighter (P_1_) and darker domains. To fulfill mechanical compatibility and charge neutrality, the dark ones must be P_2_ domains, separated from P_1_ domains by (010)-type head-to-tail (HT) DWs. The P_2_ domain also forms a larger mono-domain island embedded in the P_1_–P_2_ lamellar pattern. On the right side of the PFM image, a P_3_–P_4_ lamellar pattern is seen with the same (010)-type HT DWs. Here, the two domains share the same color, as expected, and only the DWs in between appear darker. Charge neutrality is confirmed for both lamellar patterns by the c-AFM image.

Next, we reveal some fascinating architectures of the conductive DWs. The different domain patterns on the right and left side join in the middle to form a complex DW structure: The part below the green star separates a P_2_ mono-domain island from the P_3_–P_4_ lamellar pattern, while the upper part separates the P_1_–P_2_ and the P_3_–P_4_ lamellar patterns, as also displayed schematically in Fig. 2c. The lower part shows up as lined-up bright spots in the c-AFM image, where the conductive spots are P_2_–P_3_ junctions, alternating with insulating P_2_–P_4_ segments. The P_2_–P_3_ junctions, being (1$\bar{1}$0)-type HH conductive DWs are isolated, also electrically, by (100)-type HT DWs of the P_2_–P_4_ pairs. Thus, these short P_2_–P_3_ segments can appear as conductive spots in the c-AFM image only, if extending along the [001] axis as conductive ribbons.

The upper part of the complex DW structure represents the typical 2D conductive DW sheets observed in this material. It is composed of zig-zaging P_2_–P_3_ and P_1_–P_4_ junctions, where the former and the latter are (1$\bar{1}$0)-type HH and (110)-type TT DWs, respectively. Hence, this folded sheet, best resolved in Fig. 2f, g, is built of alternating stripes running along the [001] axis with opposite bound charges. Such a sharp alternation of TT and HH DWs, which naturally leads to the formation of 2D folded conducting sheets without requiring a frustrated poling^15^, is rather unique and differs from the situation in manganites, where these two types can be adjacent only in the vicinity of vortices^18,19,47^. (Here, the notations TT, HT, and HH refer to configurations of the normal component of the polarization across the 109^∘^ DWs.) The c-AFM data in Fig. 2e also confirm that the interior of the domains and the (010)-type DWs are insulating, though the latter can gain some conductance in proximity to charged DWs. For details about DW assignment, conductivity gain of (010)-type DWs and further PFM and c-AFM images see Supplementary Notes 1–2 and Supplementary Figs. 1–3.

**Fig. 3:**
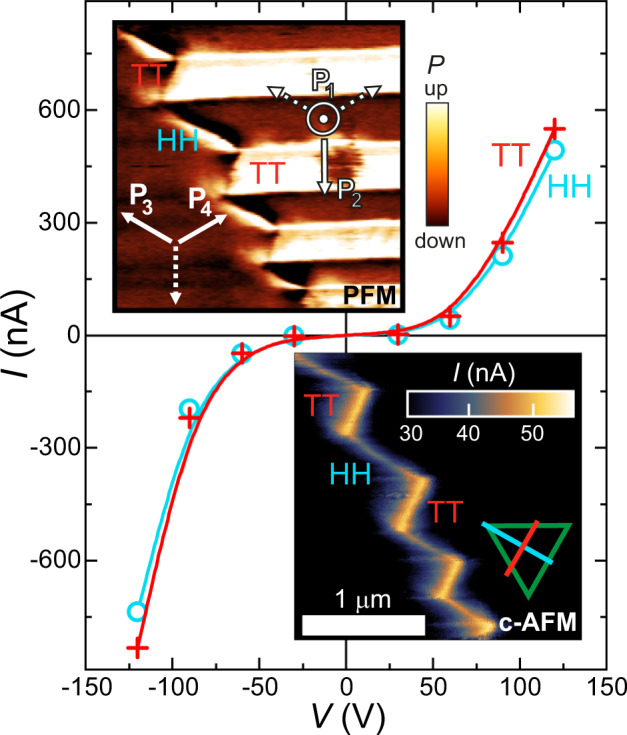
I–V characteristics of conductive DWs. I–V characteristics of HH (cyan) and TT (red) conductive DWs at 15 K. The solid lines are guides to the eye. A c-AFM image recorded at +60 V at 15 K is displayed at the bottom inset. The upper inset displays a PFM image recorded on the same area (see Supplementary Fig. 2 for an extended version of the PFM image). The arrows in the PFM image indicate the orientations of the polar axis of four polar domain states and the schematic triangle in the c-AFM image indicates the orientation of the conductive HH and TT DWs within the (111) plane.

In order to gain further insights about the charge transport in the zig-zag DWs, we studied the I–V characteristic of HH and TT segments. The PFM and c-AFM images in Fig. 3 shows a zig-zag DW formed at the interface of a P_1_–P_2_ lamella (right side) and a P_3_–P_4_ lamella (left side). Correspondingly, the zig-zag DW is composed of HH and TT segments. While both types are conductive, the conductivity of the TT DWs was found to be higher, as seen in the c-AFM image. This conductivity difference is slightly increased towards larger bias voltages, as demonstrated in the nonlinear I–V curves for both polarities. To eliminate possible artefacts in the I–V characteristic due to voltage-driven DW displacements, the data points in the I–V curve were extracted from c-AFM images recorded at different voltages, using line scans perpendicular to TT and HH DWs. While the difference between the conductivity of HH and TT DW segments was reproducible for zig-zag DWs, we could not quantify the difference between their intrinsic conductivity for the following reasons. At first, it is not clear to what extent the internal conductivity of the DWs governs the measured conductivity and how much it is affected by the tip-surface contact. Moreover, due to the close proximity of HH and TT DWs, the current path from the tip to the large-area back electrode is ill defined. Thus, the measured conductivity may represent a weighted average of the internal conductivities of TT and HH segments, rather than their individual values.

How do TT and HH DWs conduct in GaV_4_S_8_? In materials with charged DWs, the conductivity is often governed by screening charges, i.e., by mobile carriers with charge opposite to the bound charge of the DW, which are accumulated from the bulk^15,18,19,22,23,48^. If the same mechanism applies for the zig-zag DWs in GaV_4_S_8_, the conductive nature of TT and HH segments implies that both positive and negative screening charges can be accumulated from the bulk. In this scenario, the conductivity difference observed between HH and TT DWs indicates that mobile electrons and holes have different mobility. While the multivalent character of V_4_ clusters^26–32,49^ may provide an intrinsic pathway both for mobile electrons and holes, as mentioned in the introduction, the unambiguous assignment of the microscopic conductivity mechanism requires further studies. It is worth mentioning here that the strain gradient, suggested to be a driving mechanism for unconventional DW functionalities^7,9,50–52^, at the zig-zag DWs may influence the local conductivity. Nevertheless, the simultaneous conductivity of TT and HH DWs is a unique feature of this compound, as opposed to oxide ferroelectrics, where typically one type of charge carriers, either electrons or holes, are provided by defects and/or off-stoichiometry. Thus, such extrinsic mechanisms lead to an enhanced conductivity at one type of charged DW and reduced conductivity at the oppositely charged one, compared to the bulk^15,18,19,22,23,48^. In fact, the high DW mobility (demonstrated below) and the non-reproducible formation of domain patterns from cooling to cooling imply that the concentration of defects is not high in our single crystals and the physics is likely not governed by defects. The role of defects in DW pinning has been documented well in various systems^1,53–56^. In contrast, the mobility of the DWs in GaV_4_S_8_ is well exemplified by their magnetically driven displacement and erase (discussed below).

### Domain wall magnetotransport

**Fig. 4:**
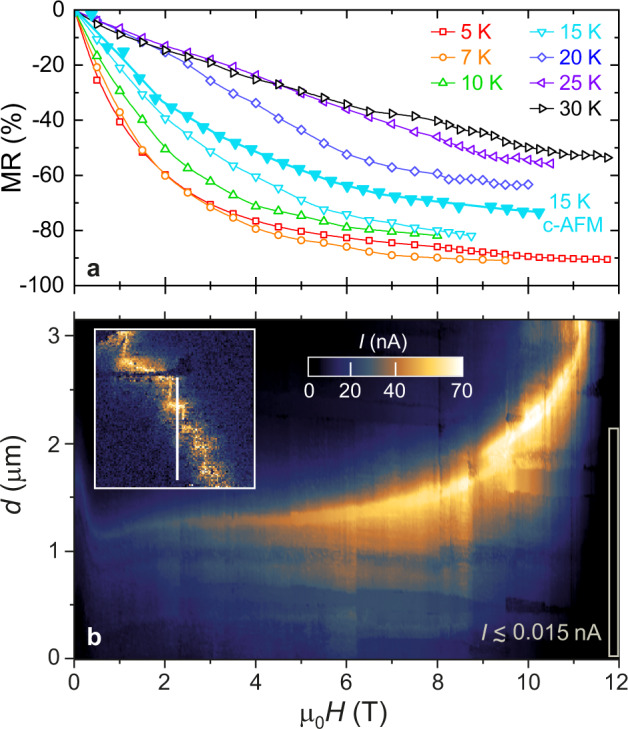
Giant negative MR of conductive DWs. **a** MR of a bulk multi-domain crystal at various temperatures, after poling with *E*_*p*_ = −5.5 kV/cm. **b** Image of c-AFM line-scan recorded while sweeping the magnetic field at 15 K. The color indicates the current for 45 V applied between the tip and the sample. The white vertical line in the inset shows the location of the line-scan in a c-AFM image recorded in zero field. The typical current at the highest fields, the region within the gray frame, is ≲15 pA. The MR deduced from the field-dependent c-AFM line scans is also displayed in (**a**).

**Fig. 5:**
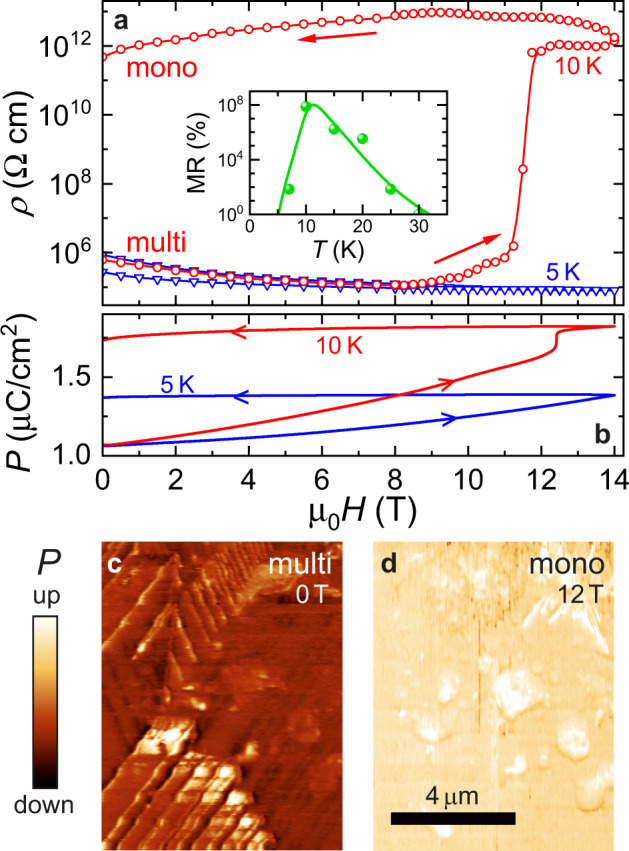
In situ magnetic erasure of conductive DWs. **a** MR of a bulk multi-domain crystal at 5 and 10 K, after poling with *E*_*p*_ = −5.5 kV/cm. Irreversible magnetic switching from a low- to the high-resistance state was observed at 10 K. The inset shows the difference of the zero-field resistance before and after the switching. **b** Field-dependent polarization of a multi-domain crystal at 5 and 10 K, after poling with *E*_*p*_ = −5.5 kV/cm. At 10 K it shows a jump at ~12 T, close to the field value where a jump in resistivity is observed. **c**, **d** PFM images recorded on the (111) surface of an unpoled crystal at 15 K in a 0 T multi-domain and the 12 T mono-domain state, respectively.

After characterizing the conductive DWs, we study the effect of magnetic field on their charge transport, which may be strong for multiferroic DWs^57–59^. Figure 4a shows the negative MR of a multi-domain crystal at various temperatures, which gradually increases with decreasing temperature and reaches ~90% in 12 T at 5 K. At temperatures between 7 and 25 K, the resistivity shows a huge irreversible jump within the field range of *μ*_0_*H* ≈ 8 − 12 T, which is shown in Fig. 5a and will be discussed later. Since the resistivity in the multi-domain state is typically 4–8 orders of magnitude lower than in the mono-domain state, the conduction is fully dominated by the DWs and the giant negative MR is solely associated with the conducting DWs. This is directly confirmed by c-AFM line scans measured while ramping the magnetic field. The tip-sample current, measured in subsequent line scans across a conductive zig-zag DW and neutral (010)-type DWs connected to it, is shown in Fig. 4b as a function of the magnetic field. The current through the conductive DW is enhanced with increasing magnetic field, as indicated by the continuous brightening of the conducting region. The enhancement of the DW conductivity translates to a negative MR as high as ~70% in 10 T, which is fully consistent with the “bulk” MR measured in multi-domain crystals (see Fig. 4a) and similar to that reported for conductive DWs in BiFeO_3_^57^. In contrast, no conductivity enhancement was observed for either uncharged (010)-type DWs or the interior of the domains. In addition to its negative MR, the conductive DW is gradually displaced by the field and exits the scanned region above 11 T, as seen in Fig. 4b. Only the magnitude of the conductivity and not the width of the conductive area around the DW is affected by the field, as becomes clear from Supplementary Fig. 4.

Does the magnetic field affect the density or the mobility of the carriers at DWs? Due to the multiferroic nature of the material, the polarization can be altered by magnetic field^35^. Since the field-driven polarization must be different for the different domain states, in principle, the charge density induced by the discontinuity of the polarization component normal to the wall could be tuned magnetically, which could charge originally neutral (010)-type DWs. For fields along the [111] axis, this effect should be the strongest for DWs involving the P_1_ domain. However, we found that P_1_–P_2_ DWs remains insulating, excluding this scenario. Thus, we believe a likely explanation of the giant negative MR is the enhancement of carrier mobility due to the suppression of spin scattering by magnetic field. Since a field-polarized FM state, with magnetization co-aligned with the magnetic field and not with the easy axes of the domains, can be achieved in GaV_4_S_8_ by fields ~1 T, the magnetic state at conductive DWs should be distinct from that in the bulk, to leave room for spin-dependent scattering in fields >1 T. Indeed, such magnetic states confined to DWs have been reported in GaV_4_S_8_^37^ and GaV_4_Se_8_^6^.

### Magnetic erase of domain walls

Besides the giant negative MR of conductive DWs, magnetic fields above a threshold value, typically *μ*_0_*H* ≥ 8 T, trigger irreversible changes in this material. This phenomenon is best seen at 10 K in the field-dependent resistivity and polarization curves respectively displayed in Fig. 5a, b. (For data at various temperatures see Supplementary Fig. 5). The resistivity shows a six orders of magnitude step-like increase at around 12 T, accompanied with a polarization jump at slightly higher fields. The sudden jumps in the two quantities, measured in subsequent poling runs, indicate the in situ magnetic switching from a low-resistance multi-domain to the high-resistance mono-domain state (For a discussion on the reversibility of this switching, see the Supplementary Note 5 and Supplementary Fig. 6). In Fig. 4b, the highly accelerated movement of the DW above 10 T also hints at a catastrophic DW expulsion event, eventually leading to the mono-domain state. In fact, the low current values (≲15 pA) detected in line scans at the highest fields roughly correspond to the typical resistance found in macroscopic transport measurements on mono-domain crystals. Moreover, at 15 K the ratio of the resistance values with the tip placed on a highly conductive DW in an unpoled sample and on the surface of a mono-domain crystal is ~10^4^, which is close to the ratio observed in macroscopic measurements at the same temperature in Fig. 2a.

PFM data in Fig. 5c, d directly support this domain switching scenario: The multi-domain pattern, observed in the unpoled crystal, is turned to a P_1_ mono-domain in *μ*_0_*H* = 12 T, which persists after switching off the field (see Supplementary Fig. 7 for c-AFM images at various in situ magnetic fields). The inset of Fig. 5a displays the magnitude of the resistivity jump versus temperature, measuring the efficiency of the in situ magnetic switching. The key role of susceptibility anisotropy in the domain control is manifested in the dramatic increase of the switching efficiency, parallel to the increase of the susceptibility, observed upon approaching the magnetic ordering temperature. (The common temperature dependence of the switching efficiency and the magnetic susceptibility can be followed in Supplementary Fig. 8a.) This observation suggests that the primary driving force for magnetic switching is the axial magnetic anisotropy, which is a consequence of the rhombohedral distortion and was found as strong as 100 k erg/cm^3^^46^, whereas higher-order magnetoelectric effects allowed by the rhombohedral symmetry play a minor role (see Supplementary Fig. 8b). We note that the control of polarization by means of magnetic anisotropy may also be viewed as an indirect, higher-order magnetoelectric effect. At low temperature, *T* = 7 K, the switching efficiency shows a fast drop, thus, deviates from the susceptibility, likely due to the reduced mobility of the DWs at the lowest temperatures. In fact, no field-induced switching could be achieved at 5 K, though the polarization gradually increases up to 14 T and stays at the highest value when reducing the field to zero. This still implies some reconfigurations of the domain structure, yet the density of conductive DWs is not reduced below the percolation limit.

In summary, we demonstrated the fascinating properties and in situ electric and magnetic control of naturally formed highly conductive DWs in a non-oxide material, the multiferroic GaV_4_S_8_. As a unique attribute of these DWs, they fully dominate the electrical transport of the material. While their emergence is witnessed by the large resistivity drop of multi-domain crystals at *T*_*JT*_, these walls can be magnetically annihilated by driving the system to the mono-domain state: Consequently, their efficient manipulation enables on-demand gigantic switching of the sample resistance by several orders of magnitude. In addition, their versatile architectures provide new routes to design and control complex networks of conductive DWs. For example, the immediate vicinity of electron- and hole-like conductive segments is not only fascinating, but also allows for the first time to design *p* and *n*-type elements in nanoscale proximity and lets us envision *p*–*n*-junction-like functional objects in non-oxide conducting DWs. These findings shall trigger an extensive search for novel non-oxide materials hosting easy-to-control topological defects and exotic electronic and magnetic states associated with them.

## Methods

### Polarization measurements

All experiments were performed on insulating single crystals of GaV_4_S_8_, which were prepared by the chemical vapor transport method, using iodine as a transport agent^43^. Electric polarization was recorded by conventional pyroelectric current measurements using a Keysight electrometer. For this purpose, a (111)-cut crystal was contacted by silver paste in a top-bottom capacitor geometry. The poling fields, either electric or magnetic, were applied along [111] direction at 50 K and the sample was cooled to 4 K, where the poling fields were switched off and the sample was kept shorted for 15 min. Pyroelectric current was measured during heating with a heating-rate of 7 K/min. Magnetic field was swept at a rate of 1 T/min when recording the magneto-current in the magnetic field-dependent polarization studies. The polarization was obtained by integrating the pyroelectric current or magneto-current over time.

### Resistivity measurements

The dc resistivity was measured by the four-probe method, where a Keithley electrometer (6517B) was used to apply a source voltage of ±8 V between terminal 1 and 2, located at opposite (111) faces of the sample, as well as to measure current through these terminals. The voltage drop between terminals 3 and 4 was recorded by a Keysight electrometer (B2987A). Terminal 3 and 4 was adjacent to and co-planar with terminal 1 and 2, respectively. Each co-planar contact pair nearly covered the corresponding surface with a small gap between them. During the poling procedures, the terminals, 1 and 3 as well as terminals 2 and 4, were shorted and the poling fields were applied from 50 K down to 4 K, where the poling fields were switched off before starting the measurements during heating the sample. For temperature- and field-dependent measurements, resistivity was recorded after stabilizing the temperature and the magnetic field in each step in order to avoid pyroelectric and magnetoelectric contributions, respectively.

### Magnetic measurements

The dc magnetization measurements were carried out with a Physical Property Measurement System (14 T—PPMS) of Quantum Design, while the ac susceptibility was measured with a Magnetic Property Measurement System (5 T MPMS—SQUID) of Quantum Design. For electric-field poling, a home-designed probe was developed for the MPMS system and the same poling protocol was followed as for the polarization measurements. The slight difference in the critical fields for the magnetic transitions in Fig. 1d, f is likely caused by slight misorientation of the samples or slight mismatch of the temperature readings of the two instruments.

### PFM and c-AFM

Cryogenic AFM measurements were performed in an attoLiquid2000 setup in an atmosphere of 20 mbar He exchange gas using conductive Pt/Ir-coated ANSCM-Pt tips. The unique cryogenic setup allows for scanning probe studies in magnetic fields up to 12 T, but only supports the detection of the out-of-plane piezoelectric coefficient. Measurements were performed on an as-grown (111)-face of the crystal, with a polished parallel (111)-face of the sample attached to a back electrode. For PFM measurements, an external Stanford Instruments SR830 lock-in amplifier was used to apply an excitation voltage of 5 V to the tip at a fixed frequency of 17.87 kHz, while the back electrode was grounded. The signal was optimized for maximum domain contrast by setting the phase shift such that all signal appears in the X-channel, when scanning the interior of the domains. PFM measurements while changing magnetic fields were performed using an Intermodulation Products Multifrequency Lock-in Amplifier in resonance tracking mode. The c-AFM measurements were performed by applying dc bias voltages of up to 60 V to the bottom electrode and detecting the current flowing through the tip with a FEMTO I/V converter. The I–V curve is obtained from c-AFM images recorded at 15 K at various voltages of up to ±120 V. The value of current at a fixed voltage for HH and TT DWs is obtained by taking the mean current value of cross-sections of fixed length across multiple DWs.

## Supplementary information

Supplementary Information

## Data Availability

The experimental data that support the findings of this paper are available from the corresponding author upon reasonable request.
