# Supplementary material for: Giant conductivity of mobile non-oxide domain walls
Source: Nat Commun. 2021 Jun 25;12:3975. doi: 10.1038/s41467-021-24160-2 (PMC8233373; doi:10.1038/s41467-021-24160-2)
Supplement: Supplementary file 1 — Supplementary Information [file 41467_2021_24160_MOESM1_ESM.pdf]

Supplementary Information for

Giant conductivity of mobile non-oxide domain walls

S. Ghara,[1] K. Geirhos,[1] L. Kuerten,[2] P. Lunkenheimer,[1]
V. Tsurkan,[1,3] M. Fiebig,[2] and I. Kézsmárki[1]

[1]*Experimental Physics V, Center for Electronic Correlations and Magnetism, University of Augsburg, 86159 Augsburg, Germany*

[2]*Department of Materials, ETH Zurich, 8093 Zurich, Switzerland*

[3]*Institute of Applied Physics, Moldova*

(Dated: May 28, 2021)

Supplementary notes:

1. Assignment of mechanically compatible DWs on the (111) plane

2. Conductivity gain of neutral DWs in proximity to a conductive DW

3. Effect of magnetic field on conductive DWs

4. Magnetic switching from multi- to mono-domain state

5. Magnetic switching from mono- to multi-domain state

6. c-AFM images in various in-situ magnetic fields

7. Magnetically induced polar-domain switching mechanism

Typeset by REVTEX

Supplementary note 1:

Assignment of mechanically compatible DWs on the (111) plane

Here we describe the intersections of all possible mechanically compatible DWs with the (111) plane, which is imaged by PFM and c-AFM. The orientation of this plane in the cubic setting is indicated schematically in supplementary figure 1a by a green triangle, together with the directions of the polarization vectors of four polar domains, P_1-P_4. Throughout the Supplementary Information, we follow the same colour convention as in the manuscript for labeling polarization vectors, domains and DWs. All uncharged and charged DWs are tabulated in the upper and lower diagonal of supplementary figure 1b, respectively[1,2]. Their intersections with the (111) plane are represented by solid lines in supplementary figure 1c, where the three grey and the six white lines

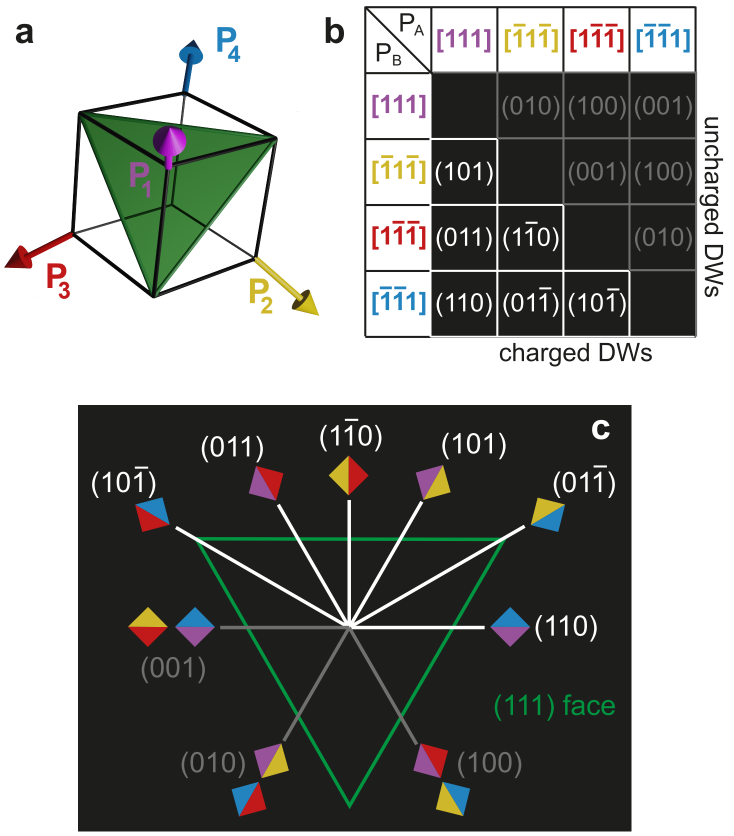

Supplementary figure 1: | **Mechanically compatible DWs and their intersections with the (111) plane. a,** Orientation of the polarization in the four polar domains, P_1-P_4, with respect to the (111) plane, which is indicated by the green triangle (reproduced from Fig. 2b of the main text). **b,** Tabulated form of all mechanically compatible DWs. The white and grey labels stand for charged and uncharged DWs, respectively. **c,** The orientation of the DWs intersecting the (111) plane. White and grey lines represent charged and uncharged DWs, respectively.

correspond to {100}-type uncharged and {110}-type charged DWs, respectively. The small bicolor squares in supplementary figure 1c indicate the adjacent domain pairs forming the corresponding DWs. The different DWs observed in the PFM and c-AFM images of the present work are assigned according to supplementary figure 1c.

The key points in assigning the domains and DWs in the out-of-plane PFM images are the following:

- The most frequent ferroelectric domain patters emerging in lacunar spinels, are mechanically compatible uncharged lamellar domain structures, where two types of polar domain states are alternating, separated by {100}-type DWs. This has been well documented already[2–4].
- When such lamellar domain structures are imaged via out-of-plane PFM on a (111) surface, there are two possible patterns observed: a) alternation of stripes with different PFM signal or b) adjacent stripes with a common PFM signal, where only the DWs in between show a contrast with respect to the internal region of the domains. The former corresponds to lamellar structures built of the unique domain state with out-of-plane polarization and one

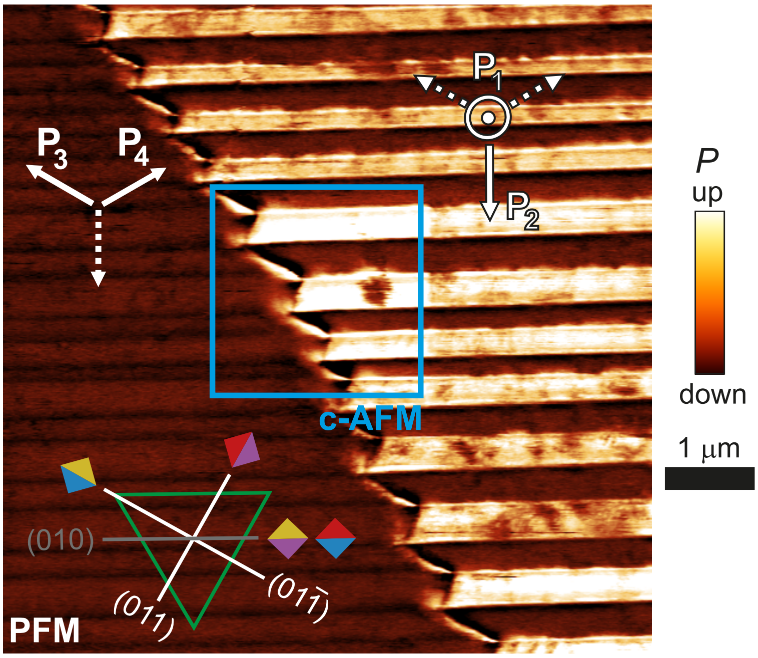

Supplementary figure 2: | **Assignment of domains and DWs in a PFM image.** An out-of-plane PFM image (extended version of the upper inset of Fig. 3 in the manuscript) showing a zig-zag DW formed at the junction of two lamellae: P_1-P_2 and P_3-P_4. The polarization directions for domains present/absent in two lamellae are indicated by solid/dashed arrows. The schematic inset at bottom indicates the crystallographic directions and assignments of the DWs observed in this PFM image.

of the other three domain states that are undistinguishable by out-of-plane PFM. The latter type is formed of two out of the three domain states that are undistinguishable by out-of-plane PFM.

- Since the DWs within such lamellar structures are mechanically compatible and uncharged {100}-type walls, from the crystallographic orientation of the DWs one can specify the two pairs of domain states (e.g.: P_1 & P_2 and P_3 & P_4), either of which can form the observed lamellar structure. From the contrast between the stripes, one can decide if the pattern is formed by the pair including the unique domain [the scenario a) above] or by the other pair [scenario b) above].

- When a more complex structure forms out of two lamellar patterns with a common orientation of the DWs within the two lamellae, one side is necessarily an a)-type lamella and the other side is a b)-type lamella, i.e. all the four domain states must build up the structure, as shown in the PFM image of supplementary figure 2.

- At the interface of the two lamellar patterns we always observed a zig-zagging wall built of two types of perpendicular {110}-type segments. For example, in the PFM image of supplementary figure 2, the zig-zag wall is composed of (011) and $(01\bar{1})$ segments. Based on the fact that both DWs are mechanically compatible, one can identify the two domain states forming the (011) segments and the other two domain states forming the $(01\bar{1})$ segments.

By this, we can make a unique assignment of all the domains within these complex structures. This assignment is fully supported by c-AFM measurements as described in the main text.

Supplementary note 2:

Conductivity gain of neutral DWs in proximity to a conductive DW

Supplementary figure 3 shows another typical example for the domain and DW patterns observed on (111) surface of GaV_4S_8 by PFM and c-AFM. The assignment of the polar domains are done in the PFM image based on supplementary figure 1, fulfilling mechanically compatibility, and based on their electrical behaviour as observed by c-AFM imaging. In order to easily follow the complex architecture of the conductive DW, the colour-thresholded version of supplementary figures 3a & 3b are shown in supplementary figures 3c & 3d, respectively. Here one can clearly observe the zig-zag 2D conducting DW, which is composed of alternating horizontal (110)-type TT and vertical $(1\bar{1}0)$-type HH charged DWs, as indicated schematically in the inset of supplementary figure 3d, implying the coexistence of hole and electron conduction channels within a 2D folded sheet. In supplementary figures 3e & 3f, we show the line profiles along the white dashed lines indicated in the PFM and c-AFM images, respectively. On the left side of supplementary figure 3e, alternating up-down peaks, indicated by different color shading, correspond to the P_1-P_2 lamellar pattern observed in the PFM image. Such an up-down peak feature on the right side of supplementary figure 3e is not observed because of the identical out-of-plane component of the polarizations of the P_3 and P_4 domains, though tiny peaks still show up at the DWs. The corresponding c-AFM line profile in supplementary figure 3f exhibits a peak at the center of conducting $(1\bar{1}0)$-type DW and it diminishes on both sides as we move far away from the conducting DW. The uncharged (010)-type DWs show weak conducting behaviour only in the close vicinity of the conducting DW. This is clearly indicated by the small peaks on the both sides of c-AFM line profile, though the coincidence of the peaks with (010)-type DWs is better seen on the left side, where the location of these DWs is obvious from the out-of-plane PFM signal. The observed conductivity gain may originate from secondary effects, such as strain.

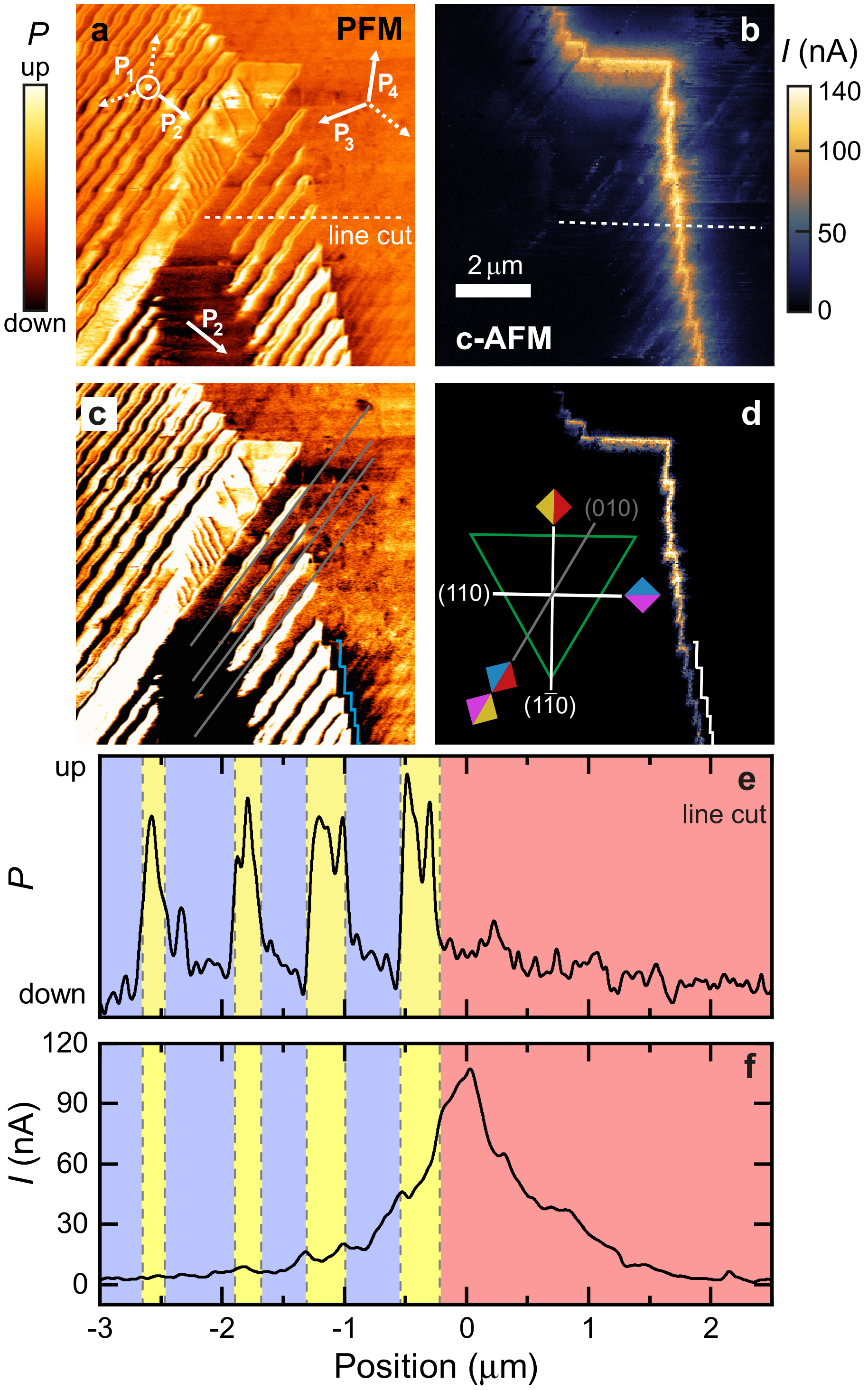

Supplementary figure 3: | **Conductivity gain of neutral DWs in proximity to a conductive folded sheet-like DW. a/b,** PFM/c-AFM images recorded on the (111) plane of a GaV_4S_8 crystal. **c/d,** The colour-thresholded view of panel **a/b**. The schematic drawing in the inset of **d** shows the orientation of observed charged (white) and uncharged (grey) DW segments. **e** & **f,** Line profiles along the dashed lines in PFM and c-AFM images, respectively.

Supplementary note 3:

Effect of magnetic field on conductive DWs

In Fig. 4b of the main text, we have demonstrated the giant MR of the conducting DWs by performing c-AFM line scans while sweeping the magnetic field. Here we reproduce the same figure (extended version) in supplementary figure 4a. Some of the line-scans (vertical cuts), indicated by dashed lines at different magnetic fields, are shown as curves with the corresponding colours in supplementary figure 4b. These line-scans data are scaled to the zero-field curve. The width of the conductivity peaks in line-scans recorded at various magnetic fields remains unchanged with increasing field, i.e. the width of the conductive region at the DW is not effected by the external magnetic field only magnitude of the conductivity. The scaling factor k, plotted in the inset, shows the field-induced enhancement of the DW conductivity.

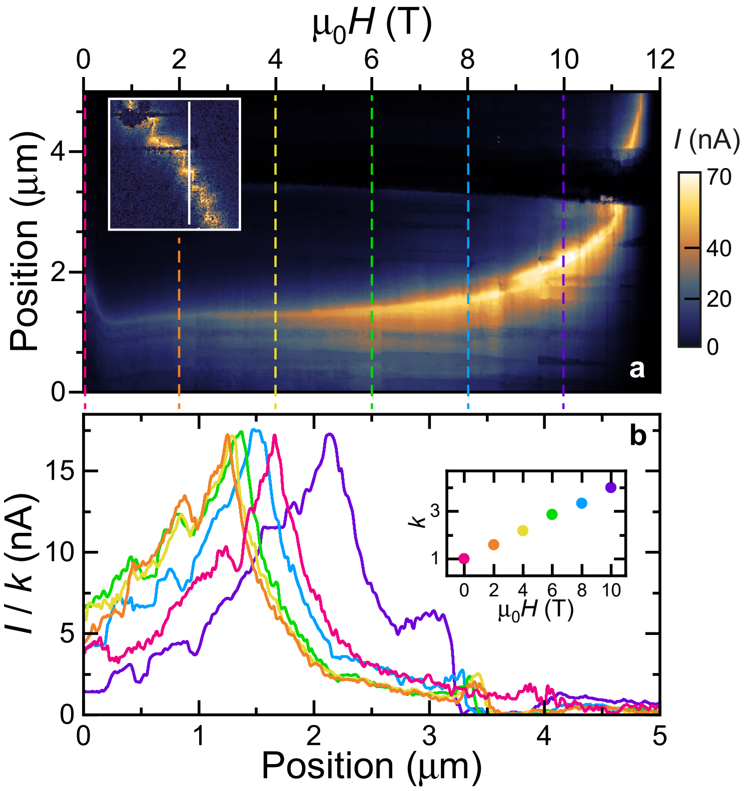

Supplementary figure 4: | **Effect of magnetic field on the width of conductive DWs. a,** c-AFM line-scan image recorded during sweeping the magnetic field at 15 K (reproduced from Fig. 3 of the main text). The white vertical line in the inset shows the location of the line-scan in a c-AFM image recorded in zero field. The black region in the upper part is a surface defect. **b,** The c-AFM line profiles recorded at different field values, as indicated by vertical lines in panel **a**. All curves are normalized to the zero-field curve. The field dependence of the scaling factor is shown in the inset.

Supplementary note 4:

Magnetic switching from multi- to mono-domain state

The magnetic field induced switching from a low-resistance multi-domain state to the high-resistance mono-domain state was investigated at various temperatures. We show the corresponding MR curves in supplementary figure 5. When approaching the low-temperature field-polarized ferromagnetic state with decreasing temperature, the switching efficiency —as measured by the relative difference of the initial and final value of the resistivity in zero field— is enhanced due to the increase of the magnetic anisotropy energy term. However, the DW mobility is expected to be reduced by lowering the temperature, which is indeed manifested in the sudden decrease of the switching efficiency below 10 K. This trend can be discerned in the inset of Fig. 4a in the main text. At 5 K no switching is observed up to 14 T.

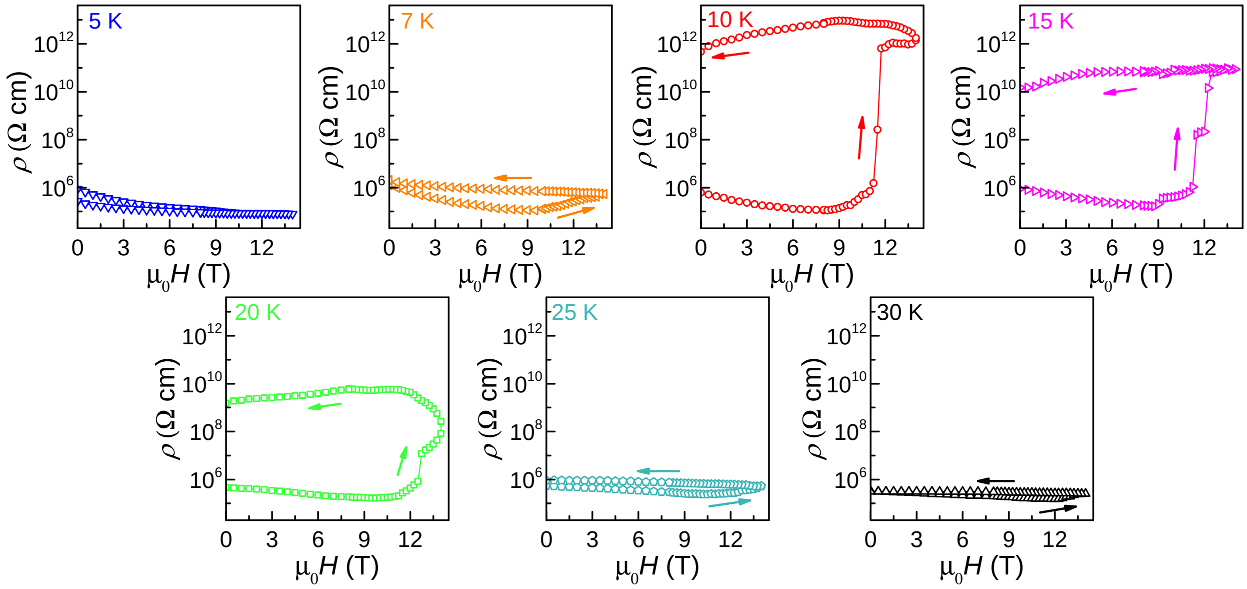

Supplementary figure 5: | **Magnetically induced switching from a multi-domain to the mono-domain state.** MR of a multi-domain crystal, created by pre-poling with E_p=−5.5 kV/cm, at various temperatures.

Supplementary note 5:

Magnetic switching from mono- to multi-domain state

Since the sign of the magnetic field does not play a role in the switching mechanism, the back switching from the high-resistance mono-domain state to a low-resistance multi-domain state cannot be achieved by the reversal of the magnetic field. An alternative mechanism for the back switching is discussed here. For this purpose, we measured the polarization (supplementary figure 6a) and the resistivity (supplementary figure 6b) with currents along the polar axis of the P_1 domain at 10 K, while rotating 14 T magnetic field in the $(1\bar{1}0)$ plane, that contains the polar axes of P_1 and P_2 domains. In an ideal case, without pinning and strain, that domain state is selected which has the largest projection of the magnetic field to its polar axis. Correspondingly, in our rotation experiments the P_1 and the P_2 states should be favoured for $0° < \phi < 90°$ and $90° < \phi < 180°$, respectively, while for $\phi = 90°$ all four domain states should be equivalent. The rotation angle ϕ is measured from the [110] axis, as shown in the inset of supplementary figure 6a. For magnetic fields in the vicinity of the polar axis of the P_1 domain state ($\phi = 35°$), the value of the polarization shows that the P_1 mono-domain state is achieved, i.e. $P = +P_s$. This is further supported by the high resistivity value observed for this angular range. Around $\phi = 90°$, where all four domains are expected to be equivalent, the simultaneous drop in the polarization and resistivity indicates the increasing population of P_2, P_3 and P_4 domain states. The fact that the polarization does not reach $-P_s/3$ for field directions close the the polar axis of the P_2 domain state ($\phi = 145°$) but is slightly negative shows that the P_1 domain state remains well populated, so that its volume fraction is $\lesssim 25\%$. In case during the rotation only the relative populations of P_1 and P_2 domains would be affected, without introducing P_3 and P_4 domains, no emergence of conducting DWs would be expected, as P_1-P_2 lamellar patterns have unchagred DWs. Therefore, the drop in the resistivity indicates that P_3 and P_4, necessary ingredients of of zig-zag conductive DWs, also emerge in the sample upon the field rotation. However, their fraction stays rather low, as the conductivity changes only two orders of magnitude, compared to the eight orders of magnitude changed observed in case of multi-domain samples with high DW density. One has to note that in the field rotation experiment the volume fractions of P_3 and P_4 domain states cannot be controlled by the field and the system could in principle avoid their emergence upon rotation. Thus, the rotation of the magnetic field, as discussed above, does not necessarily lead to a switching from the high- to a low-resistance state.

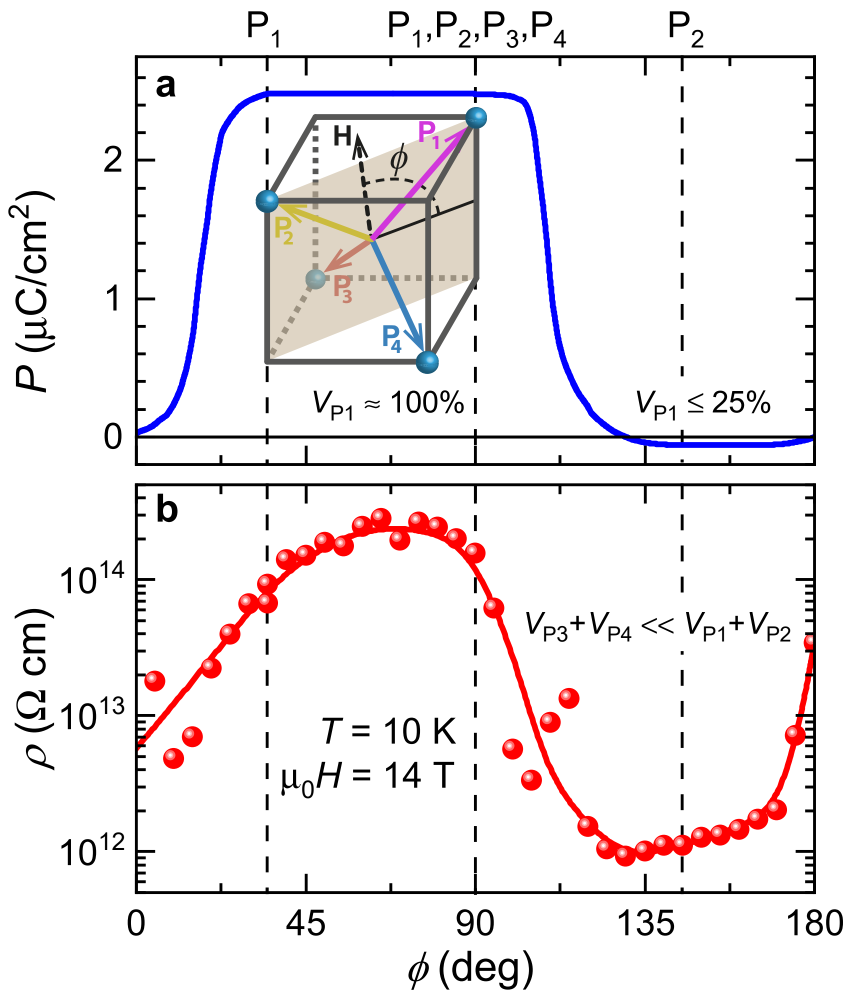

Supplementary figure 6: | **Control of domains and DWs by a rotating magnetic field. a & b**, Angular dependences of polarization and resistivity under a rotating magnetic field (14 T) at 10 K, respectively. The solid red line in **b** is a guide to the eye. The schematic in **a** shows the four possible polarization directions in the cubic reference frame. The grey-shaded plane indicates the $(1\bar{1}0)$ plane, in which the magnetic field was rotated. The labels, P_1...P_4, at the top indicate the ideal situation of the domain preference by the magnetic field during the period of rotation.

Supplementary note 6:

c-AFM images in various in-situ magnetic fields

The magnetic erase of DWs is further supported by the c-AFM images, as shown in supplementary figure 7. Since the magnetic field-dependent bulk resistivity data, shown in Fig. 5a in the main text, suggest that the sudden DW expulsion process occurs around $11 - 12\,\mathrm{T}$, which is also evidenced from the c-AFM line scan plot of Fig. 4b, we imaged the DW pattern on the (111) surface of an unpoled crystal at $15\,\mathrm{K}$ in $\mu_0 H = 10 - 12\,\mathrm{T}$. As seen clearly, the DWs are displaced slowly with increasing magnetic field from $10\,\mathrm{T}$ to $11\,\mathrm{T}$. At $12\,\mathrm{T}$, they suddenly disappear from the scanned region, which is consistent with the PFM images shown in Fig. 5c & 5d in main text, resulting in a high-resistance mono-domain state.

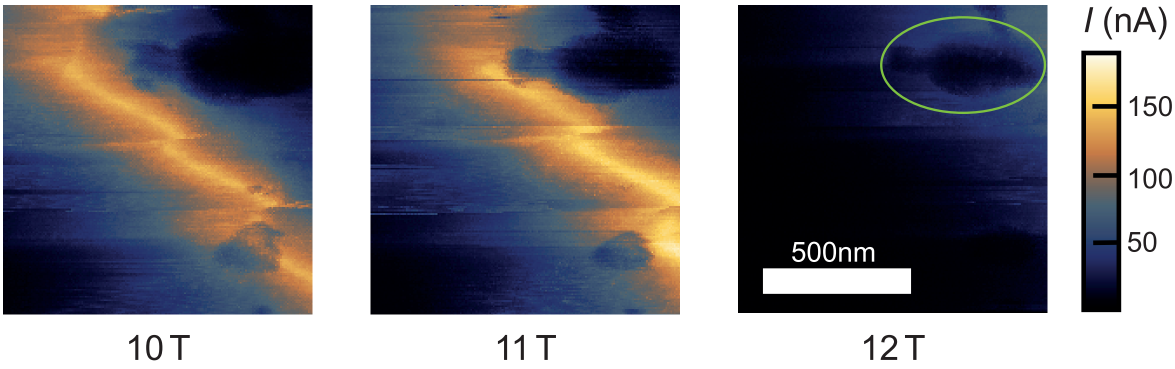

Supplementary figure 7: | **c-AFM images in various in-situ magnetic fields.** c-AFM images recorded on the (111) surface at 15 K in the presence of various magnetic fields. The green coloured frame indicates a defect on the surface.

Supplementary note 7:

Magnetically induced polar-domain switching mechanism

In the inset of Fig. 5a of main text, we have shown the temperature dependence of MR (%), representing the switching efficiency. Here, we reproduce the same figure in supplementary figure 8a (left), where we also display temperature-dependent magnetic susceptibility (right). It can be seen clearly that the temperature dependence of switching efficiency follows the same temperature dependence as the magnetic susceptibility, except for the lowest temperatures, where the switching is likely limited by decreased DW mobility. Note that the paramagnetic rhombohedral point group $3m1'$ does not allow a linear but only a quadratic ME effect. We found this higher-order ME effect is weak and its temperature dependence is opposite to that of the switching efficiency, i.e., it decreases with decreasing temperature below $T_{JT} = 45\,\mathrm{K}$. This is demonstrated in supplementary figure 8b, where we show the polarization change induced by 14 T for a mono-domain crystal at various temperatures. These results suggest that the ME effect is a secondary player and the magnetic anisotropy is the primary driving force of magnetic switching.

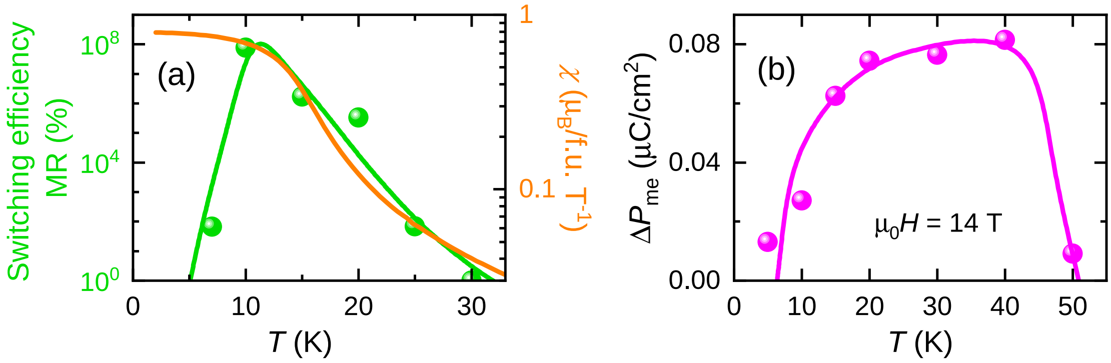

Supplementary figure 8: | **Magnetic field driven polar-domain switching mechanism.** **a**, Temperature-dependent MR (%) (left) and magnetic susceptibility (right). The susceptibility was measured at $\mu_0 H = 1\,\mathrm{T}$ applied along [111] direction. **b**, Temperature-dependent ME polarization measured in $\mu_0 H = 14\,\mathrm{T}$ for a mono-domain crystal. The solid (green and magenta) lines are guide to the eye.

[1] Erhart, J. Domain wall orientations in ferroelastics and ferroelectrics. *Phase Transitions* **77**, 989–1074 (2004).

[2] Neuber, E. *et al.* Architecture of nanoscale ferroelectric domains in $GaMo_4S_8$. *J. Phys. Condens. Matter* **30**, 445402 (2018).

[3] Butykai, Á. *et al.* Characteristics of ferroelectric-ferroelastic domains in Néel-type skyrmion host GaV_4S_8. *Sci. Rep.* **7**, 44663 (2017).

[4] Geirhos, K. *et al.* Macroscopic manifestation of domain-wall magnetism and magnetoelectric effect in a Néel-type skyrmion host. *npj Quantum Materials* **5**, 44 (2020).
